# The Hospital Penny Fund

**Published:** 1904-10-15

**Authors:** 


### HOSPITAL ADMINISTRATION.

### CONSTRUCTION AND ECONOMICS.

### "THE HOSPITAL PENNY FUND."

### EXPLOITING THE HOSPITALS FOR PRIVATE GAIN.

### A GRAVE SCANDAL.

Having failed to get together a committee of representative men as originally proposed, the promoters of this scheme, with the co-operation of *Public Opinion*, have thrown off the mask and are exploiting the hospitals, without their consent, to enable the promoters to reserve twenty-five per cent. of the receipts from the sale of the stamps for purposes in which they will participate, apart from the indirect advantage to the promoters of the advertisement. In addition to this twenty-five per cent. the scheme provides that *Public Opinion* shall secure an extra six and two-thirds per cent. of the moneys derived from the sale of the stamps. It would thus appear that thirty-one and two-thirds per cent. of the total sum realised by the sale of stamps will be deducted by the promoters and the proprietors of the newspaper associated with them. The promoters have first issued ten books of stamps in the names of the following hospitals:—Victoria Hospital for Children, St. Mary's, St. John's, Royal Free, London, German, Charing Cross, St. George's, Brompton Consumptive, and Royal Dental Hospitals. We have communicated with the authorities of these hospitals and find that they repudiate all connection with the scheme and are most anxious that it should be put an end to. In some cases the hospital authorities have referred the matter to their solicitors with this object, but, in view of the fact that the promoters announce it to be their intention to use the names of 200 reputable institutions by issuing 200 sets of hospital stamps, it is high time that the hospital authorities should meet in conference and take joint action to concert measures which shall be effective in preventing the promoters from further exploiting our great public hospitals for private profit and benefit. In publishing the letters we have received from the various hospitals we would call the attention of the Press to these replies and to the grave scandal which is here revealed, in the hope that the editors throughout the country will kindly co-operate in putting a stop to proceedings which must result in injury to the hospitals and disappointment to the public.

It should be stated that it was not untii the issue of *Public Opinion* for October 7th that the second list of the hospitals for which books of stamps were ready was published. That list is as follows:—Guy's; Epilepsy and Paralysis, Maida Vale; Sick Children, Great Ormond Street; London Lock Hospital and Rescue Home; Middlesex; National Dental; University College; Poplar; St. Bartholomew's; St. Thomas's. We would specially call the attention of everybody to the correspondence between the promoters of this scheme and St. Bartholomew's Hospital upon this point. So long ago as March 31st last Mr. Cross wrote to the promoters on behalf of the Treasurer and Almoners, declining to take any part in the scheme or to sanction it, and requesting them to withdraw the name of St. Bartholomew's Hospital, and not to circulate stamps bearing the name of the hospital. We have here evidence that the promoters are for their own purposes still using the name of the hospital where the authorities have refused their sanction, six months after this refusal had been intimated to the promoters. It therefore behoves the whole of the hospitals to at once combine and to take steps to secure that the public shall be made acquainted with the fact that "The Hospital Penny Fund" is being pushed for the benefit of its promoters against the best interests of the hospitals, the names of which are being used in defiance of the express instructions of those responsible for the conduct of their affairs. Anyone unfamiliar with the details and past history of this scheme should refer to the articles in *Truth*, May 19th and September 29th, 1904, especially, and to that in The Hospital May 14th, 1904.

### DISCLAIMERS FROM THE HOSPITALS In the Promoters' First List of Institutions.

Brompton Hospital for Consumption and Diseases of the Chest.

The Secretary writes:

"In reply to your inquiry as to the 'Hospital Penny Fund,' for which I thank you, I beg to state that the name of this hospital is not used by the sanction of this committee, as they do not wish to be identified with the fund."

October 7th, 1904. Wm. Hy. Theobald, Secretary.

Charing Cross Hospital.

The Secretary writes:

"In reply to your letter of yesterday, I beg to inform you that we have declined to have anything to do with the Penny Fund. When the article appeared in *Public Opinion* I brought it before the Board of the Hospital, who directed me to write and request that the name of our hospital might be withdrawn, as we did not approve of the undertaking, and had no desire to be connected with it in any way, and that the name of the hospital was published without authority. The Secretary of the Fund wrote a reply which has been referred to the solicitors to the hospital, and we are now awaiting their advice. I am afraid that there is no law by which they can be restrained from carrying out their project. If the Central Hospital Council would address a letter on behalf of the hospitals to the public Press denouncing the project, it might do good and suppress it. I have had a number of letters from different hospitals on the subject, and there is not one I know of who has approved the scheme."

October 6, 1904. ARTHUR READE.

German Hospital.

The Secretary writes:

"Replying to your inquiry, I have to inform you that some months ago a photograph of the hospital was furnished to the promoters of the Hospital Penny Fund, for reproduction as a stamp in connection with their scheme. However, upon reading the articles in *Truth* and other papers on the subject, the Committee decided to withdraw entirely from having anything to do with the scheme, and on the 28th ultimo I wrote to the Secretary of the Fund to that effect, and requested that the sale of stamps for this hospital should be stopped. He replied that it was impossible to comply with the request, as the books were already printed and issued. It is probable that the matter will be referred to the solicitors of the hospital."

October 7th, 1904. W. F. COCHRANE.

The London Hospital.

The following letter from the Chairman, the Hon. Sydney Holland, defines the position of this hospital:

"I see that what is called the Hospital Penny scheme is being started, despite the article in *Truth*, with a Mr. Goldman as secretary, and I see that my name is mentioned on the papers sent with it, and that the London Hospital is one of the hospitals which is to be first favoured with the results of the collection, if any.

"May I say that this has been done without consulting me as chairman, or the committee of the hospital, and that I know nothing of the people connected with the fund?"

SYDNEY HOLLAND,
September 23rd, 1904. Chairman.

The Royal Dental Hospital of London.

The Secretary writes:

"In answer to your inquiry I beg to inform you that this hospital has not given its sanction and co-operation to the Hospital Penny Fund, and by direction of our chairman I have informed the promoters of that fund that it is not the intention of this hospital to associate itself with it."

October 6th, 1904. J. FRAS. PINK.

Royal Free Hospital.

The Secretary writes:

"Replying to your inquiry respecting the Hospital Penny Fund, I have to inform you that the name of this hospital has been used in connection therewith, without the consent of the hospital authorities, and I have informed the Fund that my Board are unable to give their approval to the scheme, and desire that the name of the hospital may not be used in connection therewith.

"A letter to the above effect was addressed to the Hospital Penny Fund on the 22nd ulto."

October 6th, 1904. C. W. THIES.

St. George's Hospital.

The Secretary writes:

"I beg to acknowledge your letter of the 5th inst. In reply I beg to inform you that we do not propose to have anything to do with the Hospital Penny Fund, and that I have written to the secretary of the movement, requesting him to remove our name from his printed list, which was inserted originally without our consent."

October 6th, 1904. C. W. TODD.

St. Mary's Hospital.

The Secretary writes:

"In reply to your letter I beg to say that the name of this hospital is not being used by the promoters of the Hospital Penny Fund with the authority of our Board. The matter was discussed here this afternoon by the Finance Committee who decided to have nothing whatever to do with the concern. I have seen the references to it in *Truth* both the other day and in April, and am surprised that the latter did not finally extinguish the scheme."

October 5th, 1904. THOMAS RYAN.

Victoria Hospital for Children, Chelsea, S.W.

The Secretary writes:

"Permission was given for the name of the hospital to be used on stamps issued by the Hospital Penny Fund on the understanding that the London and other large hospitals had approved their scheme and had given similar permission. In view of the facts that have come to light since, the committee will no doubt reconsider the matter."

October 6th, 1904. H. G. EVERED.

**It will be observed that the names of eight of the ten hospitals on the first list of the promoters have been used without the sanction and in defiance of the express wish of the managers of the hospitals in question. No reply has yet been received from one hospital—St. John's for Skin Diseases.**

### In the Promoters' Second List of Institutions.

Guy's Hospital.

The Secretary to the Treasurer writes:

"In reply to your favour of the 8th inst. regarding the Hospital Penny Fund, you will see from the enclosed copied correspondence that the Governors of this hospital have not sanctioned the use of its name. We have not provided the Fund with any material, and the inclusion of the name of Guy's Hospital in the list published in *Public Opinion* is unauthorised."

C. H. WELLS,
October 10th, 1904. Secretary to the Treasurer.

The Hospital for Sick Children, Great Ormond Street.

The Acting Secretary writes:

"I beg to acknowledge the receipt of your letter of the 8th instant and to say that this letter shall be placed before the Committee of Management which meets on the 22nd instant, when the papers we have of the Hospital Penny Fund will be placed before them, together with your article in your issue of May 14th last, and the article in *Truth* of the 29th ulto."

JAMES McKAY,
October 10th, 1904. Acting Secretary.

London Lock Hospital and Rescue Home, Harrow Road, W.

The Secretary writes:

"The so-called Hospital Penny Fund are certainly not authorised to use our name in any way."

October 11th, 1904. A. W. CRUIKSHANK.

The Middlesex Hospital.

The Secretary-Superintendent writes:

"I am in receipt of your letter of the 8th instant, and in reply beg to state that the weekly Board of Governors of this hospital have declined to entertain the scheme submitted by the promoters of 'The Hospital Penny Fund,' and no authority has been given to use the name of this hospital in any manner in connection with the scheme."

F. Clare Melhado,
October 10th, 1904. Secretary-Superintendent.

North London or University College Hospital.

The Secretary writes:

"I beg leave to acknowledge receipt of your letter of the 8th instant, a reply to which I hope to send you in a few days." Newton H. Nixon,
October 10th, 1904. Secretary.

Poplar Hospital for Accidents, Poplar, E.

The Assistant Secretary writes:

"In reply to your letter of 8th inst., concerning the Hospital Penny Fund, we were asked to send the Fund a picture of this hospital for the purpose of having a stamp made. Thinking this might serve as an advertisement, we sent a photograph of the hospital, but beyond this nothing has been done, and the matter was never laid before our committee," Alfred A. Deighton.
October 11, 1904.

St. Bartholomew's Hospital.

The Clerk, Mr. W. H. Cross, writes, enclosing copy of the following correspondence:

St. Bartholomew's Hospital,
March 31, 1904.

"Your circular letter of the 16th instant, with its enclosures, has been considered by the treasurer and almoners of this hospital.

"They desire me to inform you that they do not see their way to taking any part in the proposed scheme or sanctioning it so far as this hospital is concerned; and they beg that, if you have circulated stamps bearing a view of this hospital and thus leading it to be inferred that we are parties to the scheme, you will withdraw the same and do your best to correct any misapprehension in the matter."

F. L. Rawson, Esq., (Signed) W. H. Cross,
Hospital Penny Fund, Clerk.
3 Budge Row, E.C.

St. Bartholomew's Hospital,
May 2nd, 1904.

"Sir William Treloar has informed me of his interview with you, and has handed me the book of stamps issued by you bearing a view of this hospital.

"Let me ask your attention to my letter of the 31st March last, and permit me to inquire what steps you have taken to correct the misapprehension which must be caused by your issue of these stamps, that the authorities of this hospital are parties to the scheme."

(Signed) W. H. Cross,
F. L. Rawson, Esq., 3 Budge Row, E.C. Clerk.

In reply to this letter Mr. Rawson declared:

"The steps taken to correct the misapprehension have been that I stated at the meeting of the secretaries exactly what had happened with regard to your hospital, and giving the effect of your letter of March 31st, and no more of the books have been printed. On the contrary, another book has been printed, containing the views of one of the hospitals who have sanctioned the scheme and sent the signature of their principal patron to be placed at the bottom of the stamps. I also have told everyone that I have come in contact with, interested in the matter, of the misunderstanding."

St. Bartholomew's Hospital,
September 23rd, 1904.

"In reply to your letter of the 22nd instant, I can only refer you to my letters of March 31st and May 2nd last, addressed to Mr. F. L. Rawson, informing him that the Governors of this hospital do not see their way to taking any part in the proposed scheme.

"Will you please also take this as a reply to your letter of the 21st instant, addressed to Sir William Treloar, which he has forwarded to me."

The Secretary, (Signed) W. H. Cross,
Hospital Penny Fund, Clerk.
13 Henrietta Street, W.C.

The names of the hospitals included in the promoters' second list were only known on Saturday last, and there has not been time to receive replies from all the institutions. It will be seen, however, that in the case of the seven hospitals from which replies have been received, the use of the hospital's name is unauthorised, though in one or two instances the matter has not yet been before the hospital committees.

